# Enriching Genomic Resources and Transcriptional Profile Analysis of *Miscanthus sinensis* under Drought Stress Based on RNA Sequencing

**DOI:** 10.1155/2017/9184731

**Published:** 2017-11-29

**Authors:** Gang Nie, Linkai Huang, Xiao Ma, Zhongjie Ji, Yajie Zhang, Lu Tang, Xinquan Zhang

**Affiliations:** ^1^Department of Grassland Science, Animal Science and Technology College, Sichuan Agricultural University, Chengdu, Sichuan 611130, China; ^2^Department of Agronomy, Purdue University, West Lafayette, IN 47906, USA

## Abstract

*Miscanthus* × *giganteus* is wildly cultivated as a potential biofuel feedstock around the world; however, the narrow genetic basis and sterile characteristics have become a limitation for its utilization. As a progenitor of *M.* × *giganteus*, *M. sinensis* is widely distributed around East Asia providing well abiotic stress tolerance. To enrich the *M. sinensis* genomic databases and resources, we sequenced and annotated the transcriptome of *M. sinensis* by using an Illumina HiSeq 2000 platform. Approximately 316 million high-quality trimmed reads were generated from 349 million raw reads, and a total of 114,747 unigenes were obtained after de novo assembly. Furthermore, 95,897 (83.57%) unigenes were annotated to at least one database including NR, Swiss-Prot, KEGG, COG, GO, and NT, supporting that the sequences obtained were annotated properly. Differentially expressed gene analysis indicates that drought stress 15 days could be a critical period for *M. sinensis* response to drought stress. The high-throughput transcriptome sequencing of *M. sinensis* under drought stress has greatly enriched the current genomic available resources. The comparison of DEGs under different periods of drought stress identified a wealth of candidate genes involved in drought tolerance regulatory networks, which will facilitate further genetic improvement and molecular studies of the *M. sinensis*.

## 1. Introduction

The genus *Miscanthus* is a species of promising C4 perennial nonfood bioenergy grasses for cellulosic biofuel production [1]. Specifically, *Miscanthus* × *giganteus*, a hybrid generated from a cross between tetraploid *Miscanthus sacchariflorus* and diploid *Miscanthus sinensis*, has been intensively studied in Europe and North America as a biomass feedstock [2–7]. However, it is the only genotype currently available for use in most countries by its natural sterility and a narrow genetic base [8, 9]. Furthermore, it is highly risky and genetically difficult to improve *M.* × *giganteus* through breeding, posing limitations to its biomass productivity, abiotic stress tolerance, and climatic adaptation under some extreme conditions [10–12]. As a progenitor of *M.* × *giganteus*, *M. sinensis* was widely distributed around East Asia and it was shown that abundant wild *M. sinensis* resources were distributed in China providing a comparable yield and well abiotic stress tolerance in some places [12–16].

Drought is a common environmental stress which induces adverse impacts on almost all aspects of plant development, growth, reproduction, and yield in a temperate area, and plants must adapt to this stress to survive [17, 18]. Plant drought tolerance is a complex quantitative trait, involving multiple pathways, regulatory networks, and cellular compartments [19]. Many of drought-induced or drought-repressed genes with diverse functions had been identified by molecular and genomic analysis in model plants. In *Arabidopsis*, 299 drought-inducible genes were identified through 7000 full-length cDNA microarray [20] and were classified into two groups including function proteins and regulatory proteins [21]. In rice, 73 dependable genes were confirmed which were induced by drought, high salinity, or cold stress [22]. In a comparative analysis of 73 genes with those identified in *Arabidopsis*, 51 of them performed a similar function and revealed a considerable degree of similarity of drought stress response at the molecular level. Specially, genes involved in antioxidative metabolic pathways play an important role in detoxifying reactive oxygen species that can accumulate under drought stress conditions. In addition, it is well known that the phytohormone abscisic acid (ABA) level is essential for drought stress responses. Several genes involved in ABA biosynthesis and catabolism pathway in the drought stress responses have been identified in model plant and crops. In *Arabidopsis*, the 9-*cis*-epoxycarotenoid dioxygenase (NCED) family gene *AtNCED3* transcripts are rapidly induced by drought stress, which proved that it plays a crucial role in drought stress-inducible ABA biosynthesis [23]. CYP707A3 is strongly induced by rehydration after dehydration condition, which is a major enzyme for ABA catabolism in the drought stress response [24, 25]. Significantly, the introduction of many stress-inducible genes via gene transfer resulted in improved plant stress tolerance [25–28]. In consequence, discovering differential expression genes and analyzing the functions of these genes are important to further our understanding of the molecular mechanisms of plant drought stress response and tolerance regulation and ultimately facilitate the enhancement of plant drought tolerance through genetic manipulation.

It has been well known that many wild plants show high tolerant phenotypes against abiotic stresses, such as salt, drought, and oxidative stresses [29–31]. Based on the previous tests of drought and cold tolerance of *M. sinensis* in Europe, a much broader range of adaptation than *M.* × *giganteus* was found in this diploid species [10, 32], indicating that *M. sinensis* is considered possible to breed varieties with higher tolerance for frost and drought than *M.* × *giganteus* [14]. However, gene identification and molecular mechanisms involving in drought tolerance of *M. sinensis* are not well understood. Recently, high-throughput RNA sequencing technology was proved a powerful tool for gene discovering, gene expression, and physiological and biochemical metabolism realization under abiotic stress [33, 34]. In this study, we explored differential expression gene and transcriptional profiles of *M. sinensis* under different drought stress stages based on transcriptome analysis, which contributes to well understand the change of regulatory mechanism processes and provide molecular bases for further revealing of metabolic networks associated with drought tolerance. The drought tolerance-related genes identified in this study aimed to provide varied candidate genes for *M. sinensis* genetic improvement and crop breeding.

## 2. Results and Discussion

### 2.1. Sequencing Analysis and De Novo Assembly

A total of 6 RNA samples from *M. sinensis* drought tolerance genotype “M2010228” were sequenced using the Illumina HiSeq 2000 platform, and 349,393,396 raw reads were generated. After filtering and trimming the raw reads, a total of 316,200,846 high-quality clean reads were assembled into 114,747 unigenes using Trinity [35]. The length of unigenes ranged from 200 to 12,275 nt, the average length of unigenes was 1288 nt, and the total N_50_ was 1854 nt. Compared to previous de novo assembly of *M.* × *giganteus* by using ABySS and Phrap, the contigs obtaining longer than 200 bp were greater in this study, and a contig N_50_ length was longer than that of 1459 bp. All the unigenes were divided into two classes by gene family clustering, with a total of 65,203 distinct clusters identified which contained several similar unigene sequences (more than 70%) in each cluster and the other total of 49,544 distinct singletons generated with single unigene (Table 1).

The Illumina HiSeq 2000 platform, a short-read-based technology [36], was used leading to the generation of large-scale genomic and transcriptomic data for nonmodel crops. Furthermore, high-throughput RNA sequencing (RNA-Seq) technologies are more accurate and sensitive for detecting both low and high levels of gene expression [37]. Generally, Illumina sequencing platform is more cost-effective when compared to Roche 454 sequencing [38] and has been successfully used in transcriptome sequencing of many plant species [39–42]. In this study, an average of 52,700,141 clean reads was got from each sample, in which the number of clean reads was greatly larger than that of sequencing based on the 454 platform [43], although the average length of unigene was shorter. Therefore, the results of this study not only provide additional valuable genomic resources for *M. sinensis* but also construct reference for the comparison between two sequencing methods, which could be used for further gene discovery and the identification of molecular markers.

### 2.2. Unigene Function Annotation and Classification Analysis

Unigene annotation and classification provide rich information about expression profiles and predict the potential functions of the assembly. Furthermore, various databases for annotation could shed light on intracellular metabolic pathways and biological behaviors of genes. In this study, the 114,747 obtained unigene sequences from six *M. sinensis* samples were aligned to protein databases NR, Swiss-Prot, KEGG, COG, and GO by BLASTx and nucleotide database NT by BLASTn (*e* value < 1*e*−5). Amongst them, 91,894 (80.08%) unigenes had significant hits in NT database; 84,456 (73.60%) in NR; 64,145 (55.90%) in GO; 55,807 (48.63%) in Swiss-Prot; 55,557 (48.42%) in KEGG; and 38,458 (33.52%) in COG. In total, 95,897 (83.57%) unigenes were annotated using at least one database.

**Figure 1:**
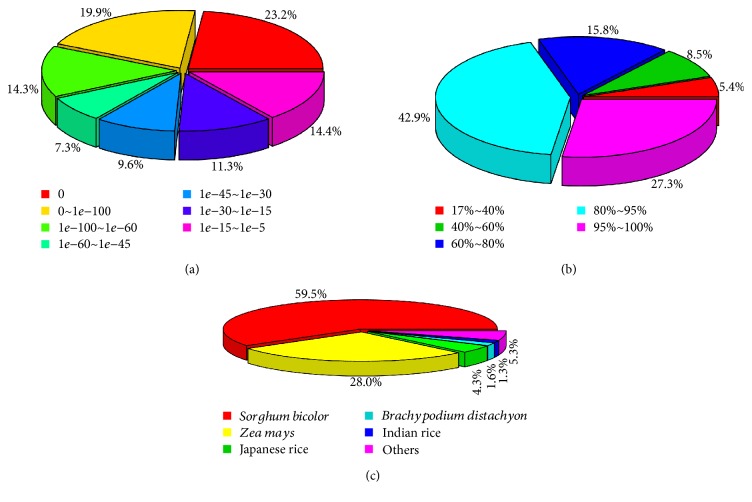
Unigene annotation results in the NR database. (a) displays the *e* value of the unigene annotation. (b) displays the identity of the similarity distribution. (c) displays the species distribution of annotated unigenes in NR database.

Within NR annotation, 74.3% of *e* value was <1*e*−30 (Figure 1(a)) and 86% of similarity distribution was >60% (Figure 1(b)). For the species distribution of all unigenes identified from six samples, the most frequent and significant annotation hits in the databases were matched to two well-annotated Poaceae plant species, including 59.5% of them which were annotated to *Sorghum bicolor* and 28.0% to *Zea mays* (Figure 1(c)). It is no surprise that the unigenes were annotated to these two species. Included within the Andropogoneae are major crops such as maize (*Zea mays* L.), sorghum (*Sorghum bicolor* L. Moench), and sugarcane (*Saccharum officinarum* L.) and species in the genus *Miscanthus*. Domesticated and wild grass species in the Andropogoneae tribe are important sources of food, feed, fiber, and fuel [44]. Previous studies showed that the high utility of sorghum as a reference genome sequence for Andropogoneae grasses was widely used for *M. sinensis* in genome-wide association analysis and QTL mapping [16, 44–46]. Swaminathan et al. [47] constructed a framework genetic map of *M. sinensis* using single-nucleotide variant (SNV) markers which were developed by deep RNA sequencing and comparison with the genomes of sorghum maize and rice (*Oryza sativa*). Ma et al. [48] created high-resolution genetic mapping of *M. sinensis* and revealed that sorghum has the closest phylogenetic relationship to *Miscanthus* by comparing the genome sequences to several grass species. Besides, the similarity of *Miscanthus* transcripts to the gene models and ESTs of sorghum, sugarcane, maize, rice, and *Brachypodium distachyon* was assessed by Barling et al. [49] and showed that a large portion of similarity was contributed to sugarcane ESTs and sorghum gene models with most matches sharing over 95% identity. In this study, the results of unigene annotation by *M. sinensis* RNA-seq showed that most of the gene annotations (59.5%) were aligned to the sorghum database, which is consistent with previous studies with the high utility of sorghum as a reference genome sequence for genus *Miscanthus*, supporting that the sequences obtained in our study were annotated properly.

**Figure 2:**
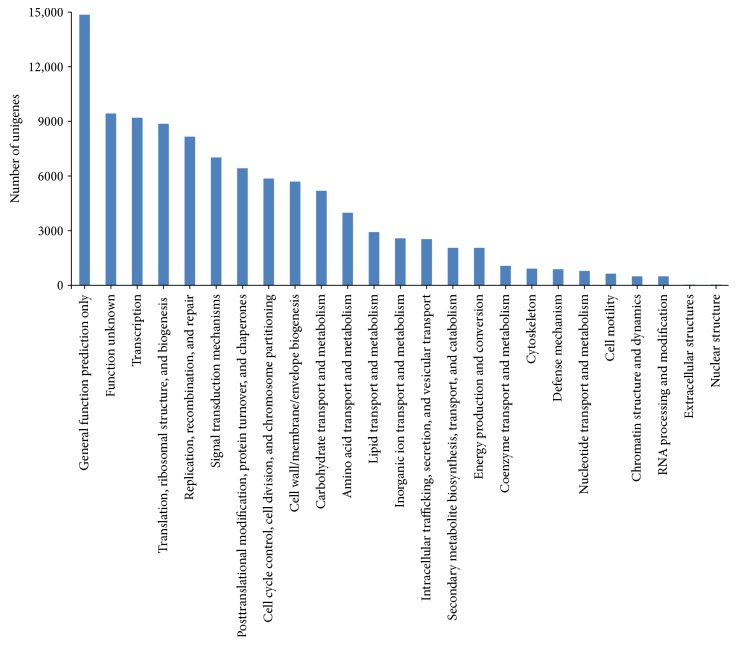
Function classification of all unigenes annotated in the COG database. A total of 38,458 unigenes were annotated under 25 function categories including (1) nuclear structure; (2) extracellular structures; (3) RNA processing and modification; (4) chromatin structure and dynamics; (5) cell motility; (6) nucleotide transport and metabolism; (7) defense mechanisms; (8) cytoskeleton; (9) coenzyme transport and metabolism; (10) energy production and conversion; (11) secondary metabolite biosynthesis, transport, and catabolism; (12) intracellular trafficking, secretion, and vesicular transport; (13) inorganic ion transport and metabolism; (14) lipid transport and metabolism; (15) amino acid transport and metabolism; (16) carbohydrate transport and metabolism; (17) cell wall/membrane/envelop biogenesis; (18) cell cycle control, cell division, and chromosome partitioning; (19) posttranslational modification, protein turnover, and chaperones; (20) signal transduction mechanisms; (21) replication, recombination, and repair; (22) translation, ribosomal structure, and biogenesis; (23) transcription; (24) function unknown; and (25) general function prediction only.

The Clusters of Orthologous Groups (COGs) of proteins were delineated by comparing protein sequences encoded in complete genomes, representing major phylogenetic lineages. Each COG consists of individual proteins or groups of paralogs from at least 3 lineages and thus corresponds to an ancient conserved domain. The results of COG analysis indicated that 38,458 unigenes were annotated under 25 categories (Figure 2), among which were mainly classified into general function prediction (14,852 unigenes, 38.6%); transcription (9175 unigenes, 23.9%); translation, ribosomal structure, and biogenesis (8864 unigenes, 23.0%); replication, recombination, and repair (8162 unigenes, 21.2%); signal transduction mechanisms (6998 unigenes, 18.2%); and posttranslational modification, protein turnover, and chaperone function (6437 unigenes, 16.7%). In addition, there still have 9442 unigenes (24.6%) classified into unknown function category indicating that the unigenes identified from *M. sinensis* transcriptome under drought stresses were very different in biological functions involving in transport and metabolism, cellular processes and signaling, and information storage and processing.

As an international standardized gene functional classification system, Gene Ontology (GO) offers a dynamic updated controlled vocabulary and a strictly defined concept to describe properties of genes and their products in any organism [50, 51]. In total, 64,145 (55.9%) unigenes were annotated to at least one of the three ontologies: molecular function, cellular component, and biological process in the GO database. In comparison, 58% of sorghum genes have GO annotation [52] as the most closely related plant to *M. sinensis*, indicating that our transcript assemblies afforded functional annotation of a comparable percentage of gene products to that of annotated plant species despite the current lack of a reference genome sequence.

**Figure 3:**
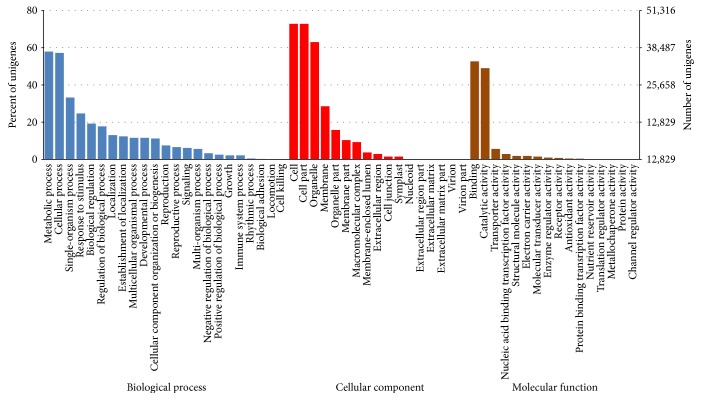
Function classification of all unigenes annotated in the GO database. A total of 64,145 unigenes were annotated under 23 different biological process categories, 17 cellular component categories, and 16 molecular function categories.

The distribution of all annotated unigenes in these three GO categories is shown in Figure 3. Among 23 different biological processes, metabolic process (57.9%), cellular process (57.0%), and single-organism process (33.2%) were the three most abundant GO categories responding to stimuli and biological regulation, suggesting active cellar and metabolic functions in *M. sinensis* leaves when exposed to drought stress. The frequent classes in cellar component were the cell part (72.9%), cell (72.9%), organelle (62.9%), and membrane (28.5%). Under the molecular function group, binding (52.5%) and catalytic activity (49.2%) were found to be the two mainly distributed categories as described, which are in agreement with the active metabolic functions in the examined tissues. With the help of GO functional classification, a large number of the unigenes were assigned to a diverse range of experimentally derived annotation. Our annotations provide a great foundation and a valuable resource for gene expression profile analysis, gene location, and gene isolation experiment in *Miscanthus* species. In addition, the main GO classifications identified through de novo transcriptome analyses in fundamental biological processes, cellar component, and molecular function were similar to previous reported studies in *M. sinensis*, *Sorghum bicolor*, [52] and *Hemarthria* [53], suggesting that our transcripts are the representative of a comprehensive *Miscanthus* transcriptome within the Andropogoneae tribe.

**Figure 4:**
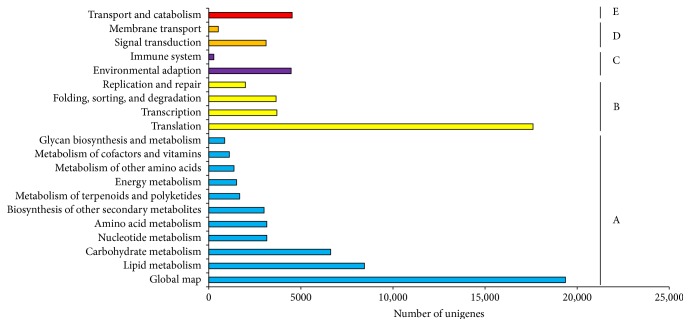
The KEGG pathway classification of all unigenes identified from six *M. sinensis* samples. A refers to metabolism category, B refers to genetic information processing category, C refers to organismal system category, D refers to environment information processing category, and E refers to cellular processes category.

The networks of gene interactions in cells could be well understood by the KEGG pathway analysis. In this study, all the unigenes were analyzed in the KEGG pathway database and 55,557 unigenes were mapped to twenty main categories including 128 different KEGG pathways (Figure 4). Most of the assigned genes were involved in a metabolism process (50,235, 90.4%), such as amino acid metabolism, carbohydrate metabolism, nucleotide metabolism, energy metabolism, lipid metabolism, and glycan biosynthesis and metabolism, suggesting a large number of genes induced by various metabolic activities under drought stress. Furthermore, a significant portion of unigenes was involved in the genetic information processing pathways (26,942, 48.5%), including transcription, translation, folding, sorting and degradation, replication, and repair. In addition, some of the unigenes were classified into organismal systems (4377, 7.9%), cellular process (4522, 8.1%), and environmental information processing (3625, 6.5%). The annotation of unigenes provides a large information base involved in drought tolerance process and plant drought response pathways, which serve an efficient guidance for future gene expression, gene network analyses, and regulatory metabolic network identification.

**Figure 5:**
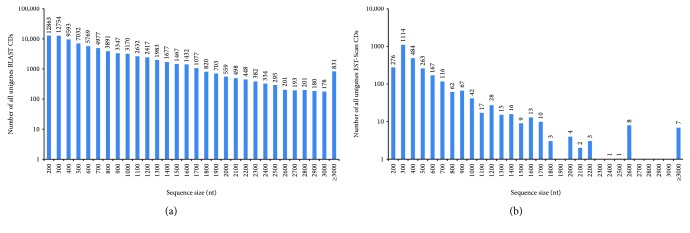
The length distribution of predicted CDs. (a) The length distribution of all unigenes BLAST CDs. (b) The length distribution of CDs scanned by EST-Scan.

In order to conduct the prediction of protein coding region (CDs), all unigenes are firstly aligned by BLASTx (*e* value < 0.00001) to protein databases in the priority order of NR, Swiss-Prot, KEGG, and COG. Proteins with the highest ranks in BLAST results are taken to decide the coding region sequences of unigenes, and the coding region sequences are translated into amino sequences with the standard codon table. Unigenes that cannot be aligned to any database are scanned by EST-Scan, producing nucleotide sequence (5′→3′) direction and amino sequence of the predicted coding region. In this study, a total of 84,633 (73.8%) unigenes were predicted to be CDs among the 114,747 assembled (Figure 5), of which 81,904 (96.8%) were aligned to the four previously discussed databases and another 2729 (3.3%) without BLAST hits were predicted by EST-Scan.

### 2.3. Differential Expression Gene Analysis

**Figure 6:**
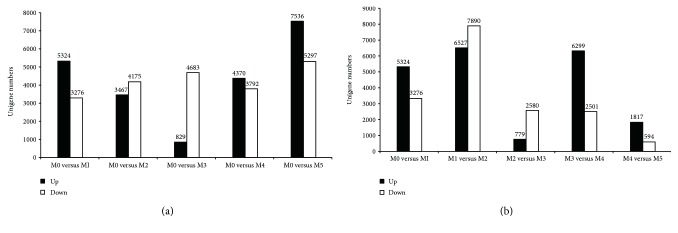
The number of DEGs at different time points under drought stress. (a) DEGs identified by the comparisons between well-watered (M0) and different drought stress treatments (M1, M2, M3, M4, and M5). (b) DEGs identified by the comparisons between two successive treatments.

A total number of 5324 upregulated and 3276 downregulated differentially expressed genes (DEGs) were detected through the transcriptome comparison of well-watered (M0) versus drought stress 5 days (M1) in genotype “M20102208.” The numbers of DEGs found in M0 versus M2 were 3467 upregulated and 4175 downregulated; in M0 versus M3, 829 upregulated and 4683 downregulated; in M0 versus M4, 4370 upregulated and 3792 downregulated; and in M0 versus M5, 7536 upregulated and 5297 downregulated (Figure 6(a)). The results showed that the number of upregulated DEGs was decreasing with the drought stress treatment time prolonging, and when the drought stress treatment continued 15 days, the upregulated DEGs just have 829. However, the number of upregulated DEGs was increased when the plant was exposed to drought tress 20 days and 30 days.

For getting the dynamic change profiles of DEGs of *M. sinensis* under drought stress, another group of comparison was conducted (Figure 6(b)). The results showed that drought stress treatments from 0 day (M0) to 5 days (M1), 5 days (M1) to 10 days (M2), and 15 days (M3) to 20 days (M4) were three critical steps with the largest change of the number of DEGs, while from 10 days (M2) to 15 days (M3) and 20 days (M4) to 30 days (M5) were relatively stable stages where the number of DEG is no obvious change. Especially from 20 to 30 days, there just a total of 2411 DEGs were found indicating that *M. sinensis* exposed to drought stress 30 days almost had no molecular regulation or growth response and plant leaves showed obvious senescence phenotype.

**Figure 7:**
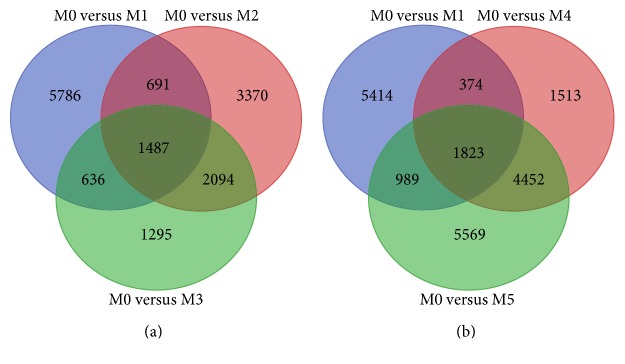
The similarity comparison of the DEGs between different time points under drought stress. (a) The DEGs identified from M0 versus M1, M0 versus M2, and M0 versus M3. (b) The DEGs identified from M0 versus M1, M0 versus M4, and M0 versus M5.

The expression variation observed from RNA-seq provides a good representation of change in transcript profiles among samples [49]. Understanding the temporal expression change of regulated genes under drought stress may give insights into the gene related with drought stress adaptation in *M. sinensis*. After the similarity comparison of the DEGs identified from various stages of drought stress treatment, the DEGs from M0 versus M1 were highly dissimilar to other comparisons (just about 25% similar), while DEGs from M0 versus M3 have 65.0% matching to M0 versus M2, and the DEGs from M0 versus M4 have 76.9% similar to those from M0 versus M5 (Figure 7). The results were highly consistent with the previous results of dynamic change comparison in which the plants exposed to the 30-day drought stress treatment were divided into three main periods, that is, 0-day to 5-day slight stress, 5-day to 15-day medium stress, and 15-day to 30-day heavy stress.

At each defined phase, the differentially expressed genes are expected to have a specific function. More significantly, there were a substantial number of genes that exhibited modulated expression under drought stress. Interestingly, for the function analysis of the upregulated DEGs among medium stress periods, we found that after the 15-day drought stress treatment, the significantly high level expressed genes were mainly functions to cytochrome c oxidase, while stag-green related gene SORBIDRAFT [54] and were commonly highly expressed after the 10-day treatment. This indicates that a larger number of genes may participate in drought tolerance regulatory mechanisms and may be responsible for the plant phenotype under drought stress. In addition, various functions related to growth and development including putative ubiquitin carboxyl-terminal hydrolase superfamily, putative AP2/EREBP transcription factor superfamily, methyltransferase ZRP4, germin-like protein subfamily, lipoxygenase, and ATP synthase were also highly expressed within the initial slight stress and heavy stress period. These results indicate that drought stress 15 days could be a critical period for *M. sinensis* drought resistant molecular mechanism regulation, and when the relative soil water content reached 51.61% at 15 days, the chlorophyll of plant leaves begins to degrade to response to the stress.

**Figure 8:**
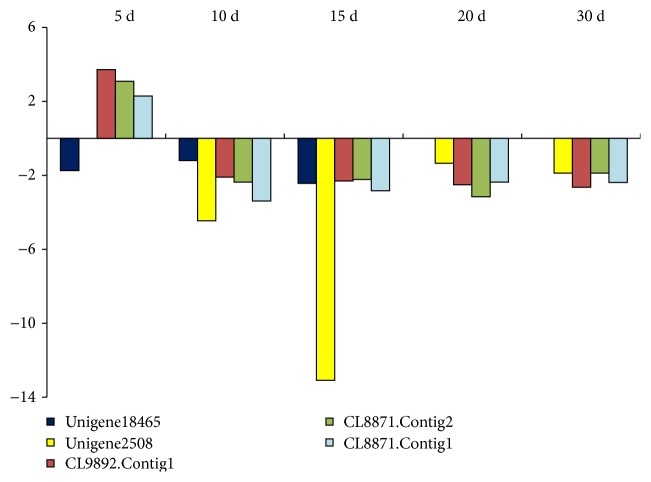
The differential expression level of key enzyme genes involved in ABA biosynthetic and catabolic pathway under drought stress. Unigene18465 and Unigene2508 functions were described as 9-*cis*-epoxycarotenoid dioxygenase 1. CL9892.Contig1, CL8871.Contig2, and CL8871.Contig1 functions were described as abscisic acid 8′-hydroxylase 1.

In addition, the endogenous ABA level in plants is essential for various ABA-dependent stress responses, especially drought and salt stresses. Recently, with the molecular basis of ABA biosynthesis and catabolism established, the increasing concern about ABA biosynthetic and catabolic enzyme gene-expressed profiles is essential for the identification of drought stress response regulatory networks. Involved in carotenoid biosynthesis pathway, 9-*cis*-epoxycarotenoid dioxygenase (NCED) is a key enzyme for ABA biosynthesis, which was originally identified from maize viviparous 14 mutants playing a crucial role in drought stress-inducible genes [55]. On the other hand, abscisic acid 8′-hydroxylase (CYP707A) is an important enzyme gene for the ABA catabolism oxidative pathway in the drought stress response, which belongs to a class of cytochrome P450 monooxygenases [24]. In this study, we focused on the dynamic expression profile of these two DEG groups described above to identify the drought stress response mechanism of *M. sinensis* (Table 2). The results showed that the NCED gene downregulated differential expression under drought stress in *M. sinensis* and the highest expressed at 15 days. The NCED transcripts are rapidly induced by drought stress to promote the ABA accumulation and to enhance drought tolerance of the plant [23]. However, among all the periods, we did not find the upregulated differential expression of NCED, indicating that the 5-day drought stress treatment in this study could be late for discovering the NCED gene in time, since the short-chain dehydrogenase/reductase (ABA2) was detected upregulated at 5 days. For CYP707A, the DEGs upregulated at 5 days and downregulated at the following periods represent that when the plant accepts the stress signal molecular, the ABA catabolism process was slow down for ABA accumulation to forcing the drought tolerance, and this could be an important mechanism for *M. sinensis* drought tolerance (Figure 8). Various crops could regulate drought stress response in different ways, although we indicate that *M. sinensis* drought tolerance was enhanced by the downregulated ABA catabolism pathway through the differential expression patterns; further studies are needed for function validation in knockout mutants or transgenic plants.

## 3. Experimental Design

### 3.1. Drought Treatment and Sample Collection

A wild drought tolerance genotype “M20102208” was collected from Sichuan Province (highway side, N 30° 08′, E 103° 14′). All the plants were propagated through rhizome division from a single individual. The plants were planted in plastic pots (20 cm in diameter and 25 cm in height) with soil mixture (50% loam with 50% fine sandy). *M. sinensis* plants were grown in a growth chamber at 30°C/25°C, 16 h/8 h (day/night), 70% relative humidity, and 500 *μ*mol photons m^−2^·s^−1^. After three-month establishment, all the replications were subjected to nature drought stress treatment. Prior to treatment, all the pots were well-watered and the soil water content (SWC) was measured by the Soil Moisture Equipment TDR 300 (Santa Barbara, CA, USA). Naturally, water stress was applied by stopping water for 30 days. Leaf samples were collected and immediately frozen in liquid nitrogen from three replications at 0- (SWC = 91.57%), 5- (SWC = 85.55%), 10- (SWC = 73.12%), 15- (SWC = 51.61%), 20- (SWC = 39.89%), and 30-day (SWC = 26.41%) drought stress treatments for RNA extraction. Total RNA was extracted using the RNeasy Plant Mini Kit (Qiagen, USA) according to the manufacturer's instructions. RNA purity, concentration, and integrity were assessed using the RNA Nano 6000 Kit for the Agilent 2100 Bioanalyzer 2100 System (Agilent Technologies, USA). After RNA isolation and quality assessment, samples were stored at −80°C until the cDNA library construction and transcriptomic assay were completed.

### 3.2. Library Construction and Sequencing

A total of 5 *μ*g of total RNA per sample was used to construct the cDNA libraries. In all, six cDNA sequencing libraries of *M. sinensis* were constructed using the NEB Next® Ultra™ RNA Library Prep Kit for Illumina (New England Biolabs, USA). Initially, the total RNA was treated with RNase-free DNase I (NEB) for 30 min at 37°C and poly(A) mRNA was isolated from total RNA using poly-T oligo-linked magnetic beads. Following purification, the poly(A)-containing mRNA was fragmented into 200–250 bp pieces using fragment buffer (Ambion), and the first-strand cDNA was synthesized using random hexamer primers and the short fragments as templates. The products were then treated with RNase H, and second-strand cDNA was synthesized by DNA polymerase I (16°C for 2 h). Finally, NEBNext Adaptor with hairpin loop structure was ligated to the cDNA and the 3′ ends of the DNA fragments were adenylated in preparation for hybridization. Subsequently, the cDNA fragments were purified and the quality of the library was evaluated using the Agilent Bioanalyzer 2100 system. The index-coded samples were clustered on a cBot System using the TruSeq PE Cluster kit v3-cBot-HS (Illumina). After generating the clusters, Illumina sequencing (paired-end technology in the Illumina HiSeq 2000 platform) of the six libraries was performed for RNA-seq analysis.

### 3.3. RNA Sequence Analysis and Drought-Induced Transcriptomic Changes

Raw reads (accession numbers SRP095822 in the NCBI SRA database) from six libraries were transformed from sequencing-received image data. Raw reads were filtered to remove those with only adapters, low quality reads, and unknown or reads with less than 20 bp in length. Following the calculation of sequence duplication, Q20, and the GC content of the clean reads, the de novo assembly of RNA-seq was conducted using Trinity (http://trinityrnaseq.github.io), which is specific for high-throughput transcript assembly of RNA-Seq data without a reference genome [35]. The unigenes were generated by Trinity modules, and the processes of sequence splicing and redundancy removing were employed. The unigenes were annotated against the following protein databases: NR (nonredundant NCBI protein sequences), KOG/COGs (Clusters of Orthologous Groups of proteins), Swiss-Prot (a manually annotated and reviewed protein sequence database), and KEGG Ortholog database using BLASTx searches (*e* value < 1*e*−5). Protein function information can be predicted from the annotation of the most similar protein in those databases. If the results of the databases conflicted with each other, a priority order of NR, Swiss-Prot, KEGG, and COG was followed when deciding the sequence direction of unigenes. The KEGG pathway database records networks of molecular interactions in the cells and variants of them specific to particular organisms. GO functional annotation was conducted with NR annotation which offers a dynamic updated controlled vocabulary and a strictly defined concept to comprehensively describe properties of genes using BLAST2GO program.

### 3.4. Differential Expression Genes and Pathway Analysis

The gene expression level was calculated by the number of uniquely mapped reads per kilobase of exon fragments per million mappable reads (FPKM) using Cufflinks (http://cufflinks.cbcb.umd.edu/). For genes with more than one alternative transcript, the longest transcript was selected to calculate the FPKM. With the expression level of each gene calculated, the differential expression analysis was conducted. The false discovery rate (FDR) as a statistical method was used to determine the threshold of the *p* value in multiple hypothesis testing, and for the analysis, a threshold of the FDR ≤ 0.001 and an absolute value of log2 ratio ≥ 1 were used to judge the significance of the gene expression differences. Tool edgeR23 was used to identify significantly up- and downregulated genes on the read count values of genes. The differentially expressed genes (DEGs) were used for GO and KEGG enrichment analyses. First, all of the DEGs were blasted in the GO database (http://www.geneontology.org/) and the gene numbers were calculated for each GO term with GO-Term Finder version 0.86 (http://search.cpan.org/dist/GO-TermFinder/). GO terms were defined as significantly enriched GO terms in DEGs, if the corrected *p* value was ≤0.05. Pathway enrichment analysis identifies significantly enriched metabolic pathways or signal transduction pathways in DEGs when compared with the whole genome background. Both GO terms and KEGG pathways with a *Q* value ≤ 0.05 are significantly enriched in DEGs.

## 4. Conclusions

High-throughput RNA sequencing technology was proved a powerful tool for gene discovering, gene expression, and physiological and biochemical metabolism realization under abiotic stress. By using the Illumina HiSeq 2000 platform to sequence *M. sinensis* under drought stress, approximately 316 million high-quality trimmed reads were generated from 349 million raw reads and a total of 114,747 unigenes were obtained after de novo assembly of the trimmed reads. Furthermore, 95,897 (83.57%) unigenes were annotated to at least one database including NR, Swiss-Prot, KEGG, COG, GO, and NT, and most of the annotations (59.5%) were aligned to the sorghum database, which is consistent with previous studies with the high utility of sorghum as a reference genome sequence for genus *Miscanthus*, supporting that the sequences obtained in our study were annotated properly. Differentially expressed gene analysis under different stress periods indicates that drought stress 15 days (soil water content reached 51.61%) could be a critical period for *M. sinensis* response to drought stress. *M. sinensis* plays an important role in improving the genetic base, abiotic stress tolerance, and climatic adaptation for this genus as a nonfood bioenergy crop. Hence, the transcriptome sequencing of *M. sinensis* reported here provides useful information for gene identification and greatly enriches the genomic available resources. The comparison of DEGs under different periods of drought stress allowed us to identify a wealth of candidate genes involved in drought tolerance regulatory networks, which will facilitate further advancements in genetic and molecular mechanisms with desired traits in further *M. sinensis* breeding programs.

## Figures and Tables

**Table 1:** The quality report of *M. sinensis* RNA sample sequencing and unigene assembling under drought stress.

Samples	TCRs	Q20 (%)	TNU	MLU (nt)	N50	DC	DS
M0	55,128,976	97.19	92,444	931	1609	42,705	49,739
M1	54,603,020	97.23	90,148	883	1570	39,303	50,845
M2	52,019,400	97.30	88,455	830	1421	38,735	49,720
M3	51,366,992	97.20	81,159	846	1473	34,424	46,735
M4	51,745,196	97.26	89,320	852	1507	38,541	50,779
M5	51,337,262	97.29	88,416	855	1480	38,862	49,554
Total	316,200,846	/	114,747	1288	1854	65,203	49,544

TCRs: number of total clean reads; Q20: percentage of bases whose quality is larger than 20 in clean reads (%); TNU: total number of unigene; MLU: mean length of unigene (nt); DC: distinct clusters, which means that there are several unigenes wherein similarity between them is more than 70% in one cluster; DS: distinct singletons, which means a single unigene comes from a single gene.

**Table 2:** Identified assembled genes involved in ABA biosynthetic and catabolic pathway under drought stress.

Gene ID	Length	Swiss-Prot ID	Description	KO definition
Unigene18465	1664	sp|O24592|NCED1_MAIZE	9-cis-epoxycarotenoid dioxygenase 1	EC: 1.13.11.51
Unigene2508	615	sp|O24592|NCED1_MAIZE	9-cis-epoxycarotenoid dioxygenase 1	EC: 1.13.11.51
CL9892.Contig1	1319	sp|Q05JG2|ABAH1_ORYSJ	Abscisic acid 8′-hydroxylase 1	EC: 1.14.13.93
CL8871.Contig2	1441	sp|Q05JG2|ABAH1_ORYSJ	Abscisic acid 8′-hydroxylase 1	EC: 1.14.13.93
CL8871.Contig1	1635	sp|Q05JG2|ABAH1_ORYSJ	Abscisic acid 8′-hydroxylase 1	EC: 1.14.13.93
